# Antiobesity Effects of Extract from *Spergularia marina* Griseb in Adipocytes and High-Fat Diet-Induced Obese Rats

**DOI:** 10.3390/nu12020336

**Published:** 2020-01-27

**Authors:** Yong-Hyun Park, Jae-Joon Lee, Hee-Kyoung Son, Bok-Hee Kim, Jaemin Byun, Jung-Heun Ha

**Affiliations:** 1 Department of Food and Nutrition, Chosun University, Gwangju 61452, Korea; yonghyunpark@hanmail.net (Y.-H.P.); leejj80@chosun.ac.kr (J.-J.L.); kyoung1033@dankook.ac.kr (H.-K.S.); kimbh@chosun.ac.kr (B.-H.K.); 2 Center for Discovery and Innovation, Hackensack Meridian Health, Nutley, NJ 07110, USA; jaemin.byun@hmh-cdi.org; 3 Department of Food Science and Nutrition, Dankook University, Cheonan 31116, Korea

**Keywords:** antiobesity, *Spergularia marina* Griseb, adipocyte, differentiation, high-fat diet, obese rat

## Abstract

Obesity has recently risen and become a serious health concern in Korea according to the westernized diet and altered lifestyle. Hence, there is a growing interest in the supplementation of phytochemicals to find a safe and effective functional ingredient to treat obesity. *Spergularia marina* Griseb (SM) has traditionally been used as a natural herb against chronic diseases in Korea. In this study, we investigated the antiobesity effects of SM in vitro and in vivo. SM ethanol extract (SME) inhibited proliferation and differentiation in murine adipocytes and primary porcine pre-adipocytes in a dose-dependent manner. In the in vivo study, supplementation of SM powder (SMP) remarkably attenuated fat accumulation in HFD-induced obese rats. In addition, SMP supplementation improved lipid profiles in the serum and tissues of high-fat induced obese rats. Collectively, these data indicated that SME exhibited antiobesity effects by modulating adipogenesis and lipolysis. Furthermore, SMP could be developed as an obesity-induced metabolic syndrome treatment.

## 1. Introduction

Obesity is characterized as an excessive accumulation of adipose tissue in the body [1]. Recently, obesity has increased and has become a serious public health and social concern in Korea [2] and in the world [3], based on the excessive caloric intake and altered lifestyle [4]. Due to rapid economic growth and dietary changes in Korea, the number of obese patients has increased [5]. Obesity is related to significant health problems due to excessive fat and related pathophysiology. Notably, obesity results in increased risks for type 2 diabetes mellitus (T2DM), cardiovascular diseases (CVD), stroke, osteoarthritis, and cancers [6,7]. At cellular level, lipid accumulation develops according to proliferation and/or *de novo* differentiation of adipocytes [8], with the accumulation of fat due to hyperplasia or hypertrophy of adipocytes [9]. The fat depot in cells is regulated by either lipogenesis or lipolysis [10]. To treat obesity, pharmacological approaches have been developed for the management of lipogenesis and lipolysis. However, these treatments inherently cause side effects, such as diabetes, CVD, cerebrovascular disease, and sleep apnea [11]. Therefore, there is a growing interest to find safer and efficacious functional ingredients to control obesity using plant-based natural sources. Various natural ingredients, crude extracts or single derived compounds, have been shown to inhibit adipocyte proliferation and/or differentiation, including *Garcinia cambogia*, chili pepper, *Panax ginseng*, brown algae, garlic, and flaxseed [12].

*Spergularia marina* Griseb (SM) is a kind of halophyte (salt-tolerant plant) that grows in mudflats at shores. In Korea, SM has been proven as an edible herb, identified to have mitigating effects against chronic disease [13]. Furthermore, the composition of SM has recently been elucidated by individual isolation [14], evaluation of physiological activity [13], and physiochemical characterization by blanching [15], demonstrating improved insulin sensitivity [16]. However, there are limited data supporting the activity of SM against excessive fat accumulation or its underlying mechanism. In this study, we investigated the antiobesity effects of SM and evaluated the potential of SM as a functional material for antiobesity. In vitro, the ethanol extracts of SM (SME) were evaluated for proliferation and differentiation in 3T3-L1 pre-adipocytes and primary porcine pre-adipocytes in a dose-dependent manner. In vivo, the effect of the SM powder (SMP) supplement was evaluated to elucidate its potential role in obesity, through body weight regulation. Furthermore, we evaluated the effect of SMP on lipid metabolism in the blood, liver, and adipose tissues in high-fat diet-induced obese rats.

## 2. Materials and Methods

### 2.1. Chemicals and Reagents

The 3T3-L1 preadipocytes were purchased from the American Type Culture Collection (ATCC). Dulbecco’s modified Eagle’s medium (DMEM), DMEM/F-12 medium, fetal bovine serum (FBS), penicillin-streptomycin (PS), and phosphate-buffered saline (PBS) were purchased from Gibco Laboratory (Grand Island, NY, USA). The 3-isobutyl-1-methylxanthine (IBMX), dexamethasone (Dex), insulin, 3-(4,5-Dimethylthiazol-2-yl)-2,5-Diphenyltetrazolium bromide (MTT), dimethyl sulfoxide (DMSO), Oil Red O, and hematoxylin and eosin (H&E) reagents were purchased from Sigma-Aldrich Co. (St. Louis, MO, USA). For in vivo analysis, triglyceride (TG), total cholesterol (TC), and high-density lipoprotein cholesterol (HDL-C) kits were purchased from Asan Pharmaceutical (Seoul, Korea).

### 2.2. Preparation of Materials

SM was obtained from Mu-An, Jeollabuk-Do, South Korea. SM was washed three times with water and dried using a salad spinner (Caous, WINDAX, Seoul, Korea). Next, SM was frozen in an ultra-freezer (MDF-U 52V, Sanyo, Osaka, Japan) at −70 °C and dried for 72 h in the freeze dryer (ED 8512, Il-shin, Yangju, Korea). Frozen dried SM was powdered using a grinder (HR2904, Philips Co., the Netherlands) and stored at −70 °C (SM powder, SMP). One hundred grams of frozen dried SMP was added to 1500 mL 80% ethanol and extracted for 3 h, three times using a heating mantle. The extract was filtered using a Whatman No. 2 paper and evaporated under reduced pressure in a vacuum rotary evaporator (EYELA VACUUM NVC-1100, Tokyo, Japan). Next, the extract was freeze-dried and stored at −70 °C (SM ethanol extract, SME).

### 2.3. Cell Culture

The 3T3-L1 pre-adipocyte cells were cultured as previously described [17]. Cells were maintained as pre-adipocytes in DMEM supplemented with 10% FBS and 1% PS. To differentiate 3T3-L1 pre-adipocytes to adipocytes, we added 10 μg/mL insulin, 1.0 μM Dex, and 0.5 nM IBMX on the first day, and only insulin was added from the second day for differentiation. Cells were incubated with 37 °C in a humidified atmosphere consisting of 95% air and 5% CO_2_. Porcine pre-adipocytes were isolated from the back (or retroperitoneal) fat tissues of 1-day males. Fat tissues were dissected and finely minced after removing all visible connective tissues. The minced tissues were incubated with collagenase (Roche, Basel, Switzerland) solution for 40 min in shaking water bath. The mixture solution was filtered through a 25 μm nylon screen (Falcon, Corning, NY, USA) and centrifugated at 1500× *g* for 10 min. Cell pellet was resuspended in Kreb’s Ringer bicarbonate buffer and centrifuged at 1796× *g* for 10 min. Next, porcine pre-adipocytes were cultured as previously reported [18]. Briefly, the number of harvested pre-adipocytes was measured using the hematocytometer and cells were plated in a dish with DMEM medium containing 10% FBS. Then, the cells were continuously incubated for 2 days with DMEM/F-12 medium containing 10% FBS, 600 ng/mL insulin, 0.1 μg/mL transferrin, 500 ng/mL hydrocortisone, and 0.05 μM rosiglitazone until the day of differentiation measurement. After the differentiation of pre-adipocytes, cells were subjected to glycerol-3-phosphate dehydrogenase (GPDH) assays, triglyceride content, and lipoprotein lipase (LPL) activity analysis on day 8.

### 2.4. Cell Viability

Cells were treated with DMSO (control) or dose-dependent SME (50–200 μg/mL) for 48 h. Cell viability was monitored using the MTT assay. The absorbance was measured at 540 nm using an ELISA microplate reader (Model 680, BioRad Laboratories Inc., Hercules, CA, USA).

### 2.5. Glycerol-3-phosphate Dehydrogenase (GPDH) Measurement

The differentiation of 3T3-L1 pre-adipocytes and the porcine pre-adipocytes was investigated by measuring of GPDH activity as previously described [19]. Briefly, the cells were washed with PBS and collected in a homogenizing buffer. After centrifugation, the supernatant was mixed with the assay buffer and substrates. The mixture solution was measured to analyze the activity of GPDH at 340 nm using an ELISA microplate reader.

### 2.6. Measurement of Lipoprotein Lipase (LPL) Activity

The determination of LPL activity was performed as previously reported [20]. One unit of LPL activity was defined as the release of 1 μmol of free fatty acid in 1 h. Glycerol stabilized 3H-triolein emulsion was used as the substrate for the measurement.

### 2.7. Quantification of Triglyceride (TG) Contents

To analyze the content of cellular TG, cells were washed with PBS and sonicated. The supernatant was collected by centrifugation at 1792× *g* at 4 °C after incubation on ice for 30 min. The supernatant was mixed with the free glycerol assay reagent in a 96-well plate. After incubation for 5 min at 37 °C, the absorbance of the solution was measured at 540 nm using a microplate reader.

### 2.8. Animal Experiments and Diets

All animal studies were approved by the Chosun University Institutional Animal Care and Use Committee (C IACUC 2014-A0029). Sprague–Dawley male rats (*n* = 32, 5 weeks old) were purchased from Orient Bio, Inc. (Seongam-Si, Korea). The animals were housed at a temperature of 18 ± 2 °C, a humidity of 55% ± 5%, and with a 12 h light–dark cycle (08:00–20:00) at the Center for Animal Experiment. The animals had access to water and food ad libitum. After 1 week of an adaptation period, the rats were randomized into four groups (*n* = 8 per group). The rats in each group were fed a normal diet (ND, 31% of calories as fat), a high-fat diet (HFD, 46%–48% of calories as fat), HFD with SMP low supplement (HFD-SL, HFD + 3% of SMP), or a HFD and with SMP high supplement (HFD-SH, HFD + 5% of SMP) for 4 weeks. The composition of the experimental diet is shown in Table 1. Body weight (BW) and food intake were measured once a week. The food efficiency ratio (FER) was calculated as FER = (total BW gain/total food intake). At the end of the study, blood samples were collected from the inferior vena cava and centrifuged at 1150× *g* for 20 min, and a supernatant (serum) was obtained. The liver and adipose tissues were harvested and weighed after washing with 0.9% saline. Tissue and serum samples were preserved at –80 °C until analysis.

### 2.9. Biochemical Analysis of Serum Samples

The activities of aspartate aminotransferase (AST), alanine aminotransferase (ALT), alkaline phosphatase (ALP), lactate dehydrogenase (LDH), TG, total cholesterol (TC), HDL-C, and glucose (Glu) levels in serum were measured using the Chemistry Analyzer (Fujifilm Dri-C hem 3500i, Fujifilm, Tokyo, Japan). The value of low-density lipoprotein cholesterol (LDL-C) was calculated using the Friedwald formula [21] (LDL-C = TC – (HDL-C – TG/5)). Atherogenic index (AI) was calculated using the formula ((TC – HDL-C)/HDL-C) and cardiac risk factor (CRF) was obtained using (TC/HDL-C) [22].

### 2.10. Lipid Contents of Liver and Adipose Tissues

Lipids were extracted from ~0.1 g of the liver and adipose tissues as described previously [23]. Briefly, the tissues were homogenized in a chloroform/methanol (2:1 v/v) mixture and centrifuged at 1150× *g* for 20 min. TG and TC were measured from the lower layer (lipid abundant) using previously reported methods [24,25]. 

### 2.11. Histological Analysis

Liver tissues were fixed with 4% paraformaldehyde and washed three times with PBS containing 0.1% Triton X-100. The tissues were embedded in O.C.T (Sakura, Japan) and the embedded block was sectioned using a Cryo-cut microtome (General Data Healthcare, Cincinnati, OH, USA) at 3–4 μm thickness. The sections were stained with the Oil Red O solution. The stained sections were observed under the light microscope (Zeiss Axioskop, Carl Zeiss, Inc., Jena, Germany). Staining area in tissues was evaluated using color-based threshold by Image J program (NIH, Bethesda, MD, USA). The total percentage of the staining area (lipid accumulation) was calculated as the sum of red-stained area divided by total area of microscopic field. Epididymal adipose tissues were fixed with 10% formalin and embedded in paraffin. The tissue blocks were sectioned at 8–10 μm and slide samples were obtained. The slides were stained with the H&E solution (Sigma, St. Louis, MO, USA) and observed under the light microscope. Adipocyte size was measured using Image J (NIH, Bethesda, MD, USA).

### 2.12. Statistical Analysis

The experimental data were analyzed using one-way analysis of variance (ANOVA) followed by Tukey’s post hoc test (GraphPad PRISM 8, San Diego, USA); *p* < 0.05 was considered statistically significant. Data are stated as mean and standard deviation (SD). 

## 3. Results

### 3.1. Effects of SME on Proliferation and Differentiation of 3T3-L1 Cells

**Figure 1:**
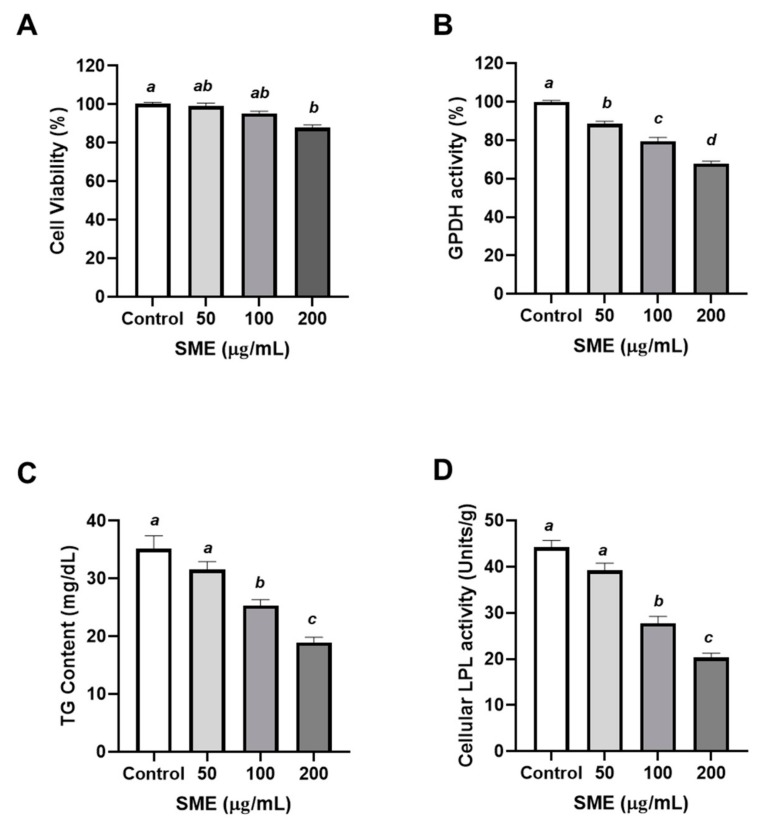
Effects of ethanol extract of *Spergularia marina* (SME) on proliferation and differentiation of 3T3-L1 cells. The 3T3-L1 pre-adipocytes were treated with SME (50, 100, and 200 μg/mL) for 48 h. Dimethyl sulfoxide (DMSO) was used as control. (**A**) Cell proliferation was determined by 3-(4,5-dimethylthiazol-2-yl)-2,5-diphenyltetrazolium bromide (MTT) assay. (**B**) Adipocyte differentiation was accessed by glycerol-3-phosphate dehydrogenase (GPDH) measurement (**C**) Triglyceride (TG) content was determined by manual methods. (**D**) Lipoprotein lipase (LPL) activity was assayed using the Nilsson-Ehle and Schotz methods. Values are means ± SDs, *n* = 3. Data were analyzed by one-way ANOVA using Kruskal–Wallis analysis followed by Tukey’s post hoc test. Labeled means without a common letter differ, *p* < 0.05.

To study the effects of SME on adipocyte proliferation, the number of cells were measured to assess the proliferation of 3T3-L1 pre-adipocyte after SME treatment at 50, 100, or 200 μg/mL 48 h later. The cell viability was over 80% under overall SME treatment (Figure 1A). SME treatment at 100 and 200 μg/mL demonstrated a tendency to inhibit cell proliferation of 3T3-L1 in comparison to the cells treated with 50 μg/mL of SME. (Figure 1A). To understand the effects of SME on adipocyte differentiation, the activity of GPDH was determined in 3T3-L1 with SME treatment at 50, 100, or 200 μg/mL. GPDH is highly expressed in mature adipocytes and its activity routinely represents adiposity in vitro [26]. SME significantly inhibited the GPDH activity in a dose-dependent manner (11.42%, 20.53%, and 32.05% reduction with 50, 100, and 200 μg/mL of SME treatment, respectively) (Figure 1B). GPDH plays an important role in the anabolic process of triglyceride (TG) formation [27] and accumulation, which are notable physiological markers of adipocyte differentiation [28]. Therefore, the TG content in adipocytes is crucial to understand the anabolic process of adipocytes, such as development and maturation. As expected, TG accumulation was significantly attenuated by ≥100 μg/mL of SME treatment (Figure 1C). The attenuation of anabolic outcomes by SME treatment inhibited the differentiation of murine pre-adipocytes. In addition, lipoprotein lipase (LPL) modulated the removal of TG from the serum and its transfer to tissues such as the liver or white adipose tissue (WAT). Furthermore, LPL activity was elevated according to adiposity. SME treatment significantly inhibited LPL activity along with the pattern of TG accumulation (Figure 1D). Collectively, SME treatment inhibited the proliferation and differentiation of 3T3-L1 pre-adipocytes.

### 3.2. Effects of SME on Proliferation and Differentiation of Porcine Pre-Adipocytes

**Figure 2:**
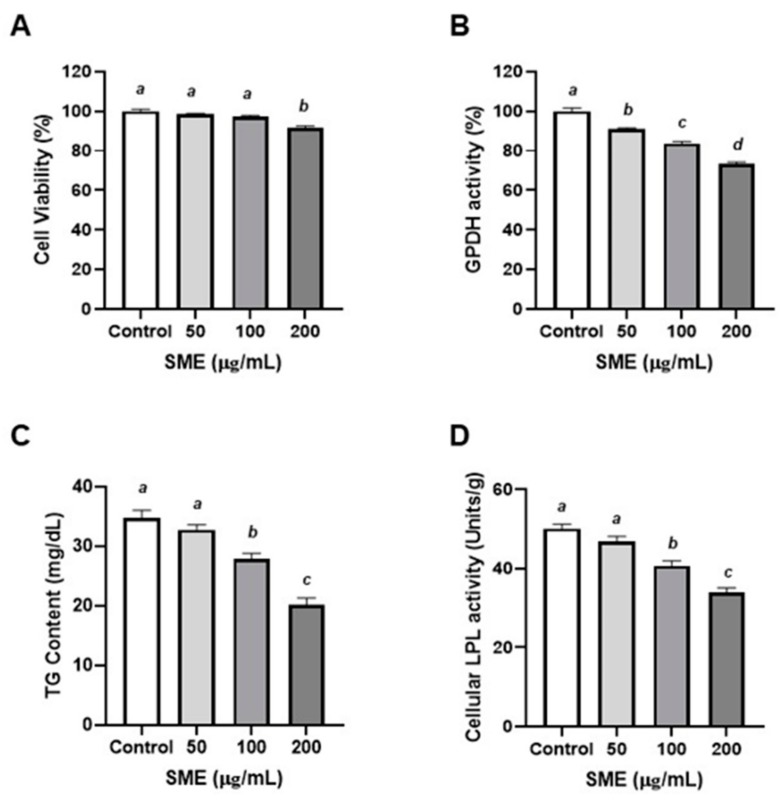
Effects of ethanol extract of *Spergularia marina* (SME) on proliferation and differentiation of porcine pre-adipocytes. Porcine primary adipocytes were treated with SME (50, 100, and 200 μg/mL) for 48 h. Dimethyl sulfoxide (DMSO) was used as control. (**A**) Cell proliferation was determined by 3-(4,5-dimethylthiazol-2-yl)-2,5-diphenyltetrazolium Bromide (MTT) assay. (**B**) Adipocyte differentiation was accessed by glycerol-3-phosphate dehydrogenase (GPDH) measurement (**C**) Triglyceride (TG) content was determined by manual methods. (**D**) Lipoprotein lipase (LPL) activity was assayed using the Nilsson-Ehle and Schotz methods. Values are means ± SDs, *n* = 3. Data were analyzed by one-way ANOVA using Kruskal–Wallis analysis followed by Tukey’s post hoc test. Means labeled without a common letter differ, *p* < 0.05.

Based on the results in murine adipocytes, porcine pre-adipocytes were also employed. It is well known that pigs are closer phylogenetically and anatomically to humans in terms of energy metabolism and storage [29]. To examine the effect of SME on the proliferation of porcine pre-adipocytes, porcine pre-adipocytes were treated with SME (50, 100, or 200 μg/mL). The 200 μg/mL SME treated group significantly impeded cell proliferation after 48 h (−9.62%) in comparison to the control group (Figure 2A). To investigate the effects of SME treatment on differentiation of porcine pre-adipocytes, the activity of GPDH was measured. SME treatment significantly decreased the activity of GPDH in a dose-dependent manner (9.13%, 16.22%, and 26.73% reduction with 50, 100, and 200 μg/mL of SME treatments, respectively) (Figure 2B). These results exhibited a similar trend as that observed in murine adipocytes (Figure 1A,B). To test the inhibitory effect of SME on adipogenesis in porcine pre-adipocytes, TG contents and LPL activity were measured. SME treatment significantly inhibited TG accumulation at ≥100 μg/mL (Figure 2C). In addition, SME treatment significantly inhibited LPL activity at 100 and 200 μg/mL doses (Figure 2D). Hence, these results indicated that SME inhibited the proliferation and differentiation of porcine pre-adipocytes. SME treatment suppressed adiposity in mammalian adipocytes, both murine and porcine (Figure 1 and Figure 2).

### 3.3. SMP Ameliorated HFD-Induced Obesity

**Figure 3:**
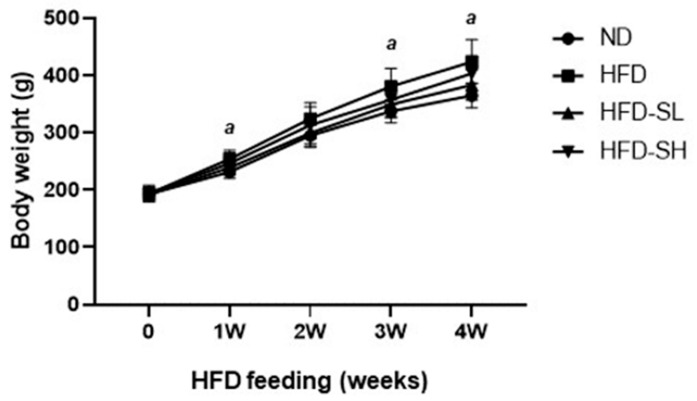
Effect of *Spergularia marina* powder (SMP) supplementation on body weight gain under high-fat diet (HFD) feeding. Rats were fed a normal diet or high-fat diet with/without the *Spergularia marina* Griseb powder supplement for 4 weeks. The rat body weights were measured every week. ND, Normal diet; HFD, High-fat diet; HFD-SL, High-fat diet + 3% of *Spergularia marina* Griseb; HFD-SH, High-fat diet + 5% of *Spergularia marina* Griseb. Values are means ± SDs, *n* = 8. Data were analyzed by one-way ANOVA using Kruskal–Wallis analysis followed by Tukey’s post hoc test. ^a^ Different from the other dietary groups at that age, *p* < 0.05.

To evaluate the effect of SM in vivo, a diet-inducible obese model was used in rats. Rats in each group were fed a normal diet (ND, 31% of calories from fat), a high-fat diet (HFD, 46%–48% of calories from fat), HFD with SMP low supplement (HFD-SL, HFD + 3% of SMP), or a HFD and with SMP high supplement (HFD-SH, HFD + 5% of SMP) for 4 weeks. The composition of ND and HFD is described in Table 1. The BW significantly increased in the HFD group compared to the ND group 1, 3, and 4 weeks after HFD feeding (Figure 3). BW gain (the BW difference between the endpoint and the initial point) in the HFD group was significantly greater than in the ND group, and the SMP supplemented groups (HFD-SL and HFD-SH) reported a significant reduction in BW gain (Table 2). The SMP supplement groups (HFD-SL and HFD-SH) demonstrated a significant decrease in food intake, indicating that the observed reduction in BW gain may be due to reduced caloric intake (Table 2). According to the FER assessment, HFD feeding (HFD, HFD-SL, and HFD-SH) demonstrated significantly higher FER levels in comparison to the ND group. However, there were no significant changes induced by the SMP supplement in the HFD-induced obese rats (Table 2). Here, it could be postulated that SMP does not inhibit the increased FER to reduce BW. To examine the weight change in organs in HFD-induced obese rats, the representative metabolic organs, such as liver and several kinds of WATs, were weighed. The liver weight was not altered by dietary intervention with SMP (Table 3). Among the WAT tissues, the weight of the epididymal adipose tissues (EAT) significantly increased in the HFD group compared to the ND group. In addition, perirenal adipose tissue (PAT) increased by HFD feeding without effect of SMP by dietary supplementation. On the other hand, the weight of other adipose tissues, such as mesenteric and retroperitoneal adipose tissues (MAT and RAT), was not significantly altered following the HFD feeding. The total adipose tissue weight demonstrated a significant increase after HFD feeding and reflected the increased EAT and PAT weights (Table 3).

### 3.4. ALT, AST, ALP, and LDH Activities in Serum

To evaluate the effect of SMP on hepatic functions 4 weeks after HFD feeding in rats, the serum levels of AST, ALT, ALP, and LDH were examined. (Table 4). All values in the HFD group were significantly higher than that in the ND group. Elevated AST levels induced by HFD feeding were significantly inhibited in the SMP supplement groups (HFD-SL and HFD-SH). The elevation of ALT was significantly inhibited in the HFD-SH group compared to the HFD group. Notably, the ALT levels (38.75 U/L) in the HFD-SH group were similar to levels observed in the ND group (30.75 U/L). Collectively, SMP supplementation remarkably decreased hepatic fat accumulation.

### 3.5. Lipids Profiles in Serum

HFD increases TC and lipid levels and it is one of the leading causes of CVD [30]. To elucidate the risk of metabolic syndrome by HFD-induced obesity, changes in the serum levels of TG, TC, LDL-C, HDL-C, Glu, and AI for atherosclerosis risk and CRF for cardiac dysfunction were assessed in obese rats. The HFD group demonstrated significantly higher levels of TC than the ND group. TC levels indicated a trend to decrease following SMP supplementation compared to the HFD group. In the HFD-SH group, the TG level was maintained similar to the level observed in the ND group (Table 5). Increased LDL-C levels in the HFD group were significantly decreased following SMP supplementation in the HFD-SH group. (Table 5). Glu levels were increased by HFD and SMP supplementation (HFD-SL and HFD-SH) inhibited the increased Glu levels in a dose-dependent manner. Based on the lipid profiles, AI and CRF were significantly elevated in the HFD group compared to the ND group; both indexes were significantly decreased with SMP supplementation (HFD-SL and HFD-SH) (Table 5). These data suggested that the SMP supplement improved lipid profiles and CVD risk in the HFD-induced obese rats.

### 3.6. TG and TC Levels in Liver and WAT

Next, we measured TG and TC levels in the liver and WAT of HFD-induced obese rats. In the liver tissue, TG and TC levels were increased in the HFD group compared to the ND group. The lower supplement of SMP (HFD-SL) significantly decreased hepatic TG levels (Table 6). TG and TC levels in EAT and MAT increased under HFD feeding; dietary intervention with SMP significantly inhibited TG accumulation in the HFD-SL group in EAT (Table 6). In addition, the high dose supplement of SMP (HFD-SH) significantly reduced TG and TC levels in MAT (Table 6). 

### 3.7. Histological Analysis in Liver and Epididymal Adipose Tissues

**Figure 4:**
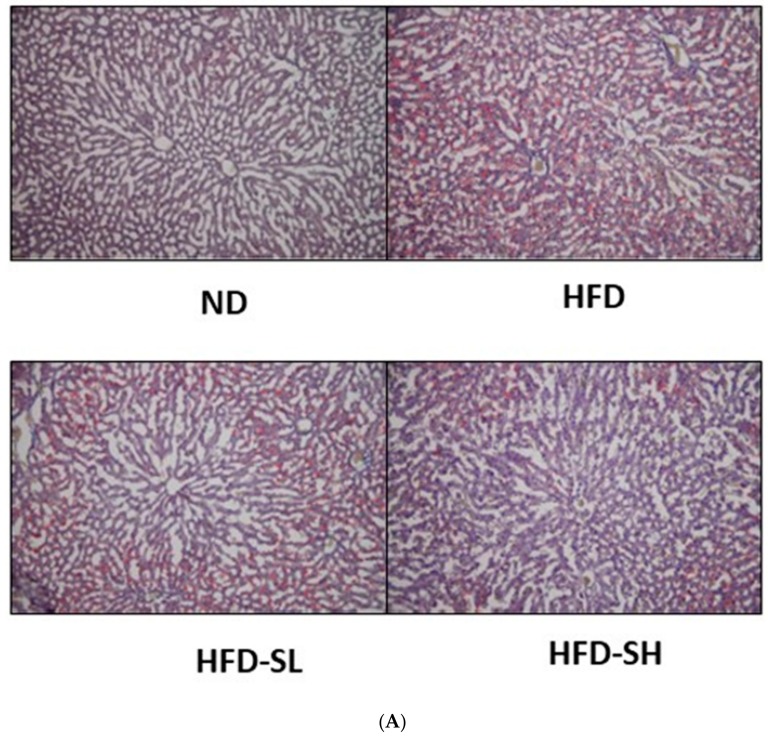

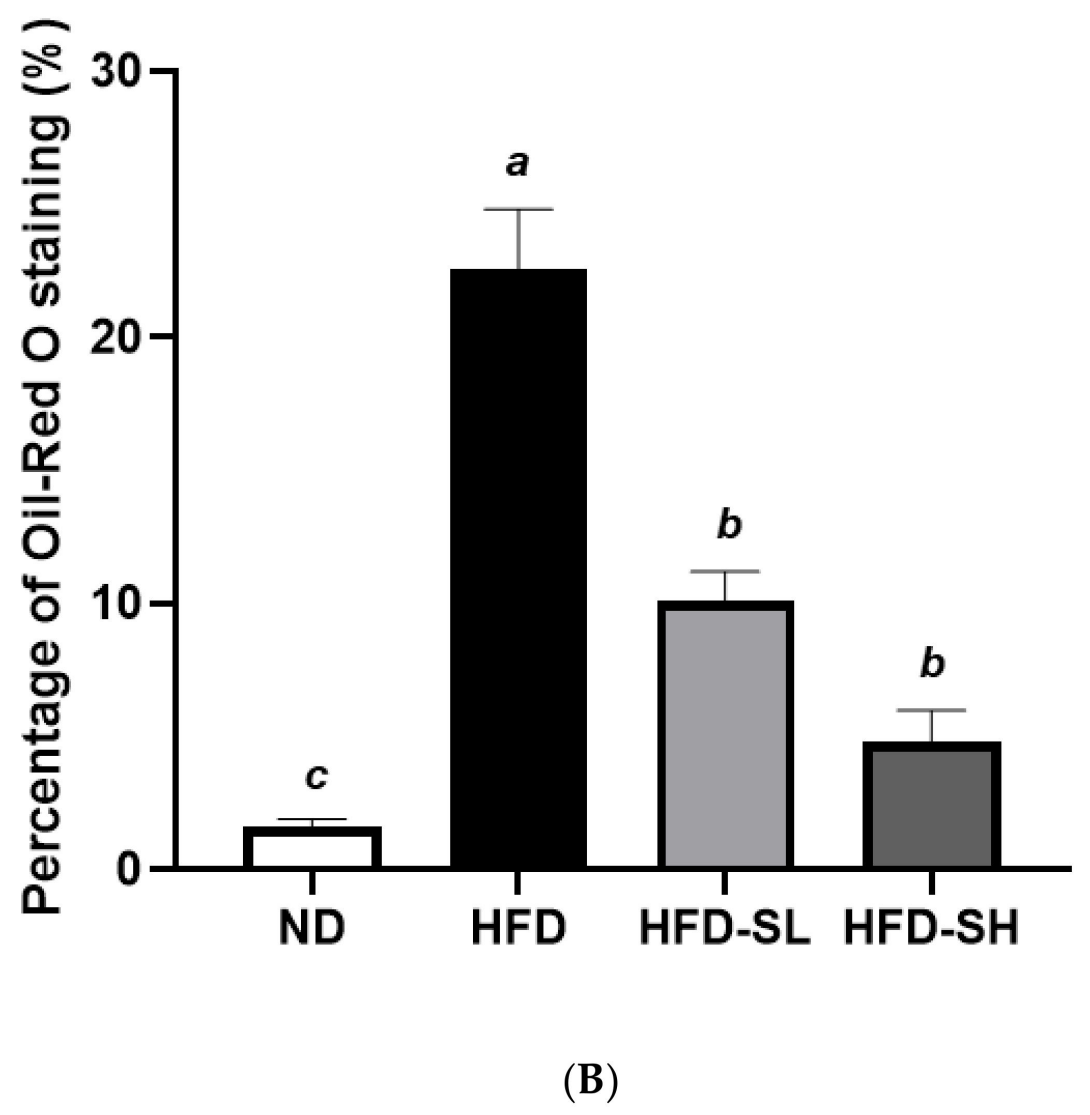
Effect of *Spergularia marina* powder (SMP) supplementation on lipid accumulation in the liver. Livers from each experimental group were harvested, fixed, and stained with Oil Red O. ND, Normal diet; HFD, High-fat diet; HFD-SL, High-fat diet + 3% of *Spergularia marina* Griseb; HFD-SH, High-fat diet + 5% of *Spergularia marina* Griseb. HFD induces numerous and larger lipid droplets; SMP supplementation inhibits lipid accumulation. (**A**) Representative figures from each group. (**B**) The quantification of A. Values are means ± SDs, *n* = 8. Data were analyzed by one-way ANOVA using Kruskal–Wallis analysis followed by Tukey’s post hoc test. Means labeled without a common letter differ, *p* < 0.05.

**Figure 5:**
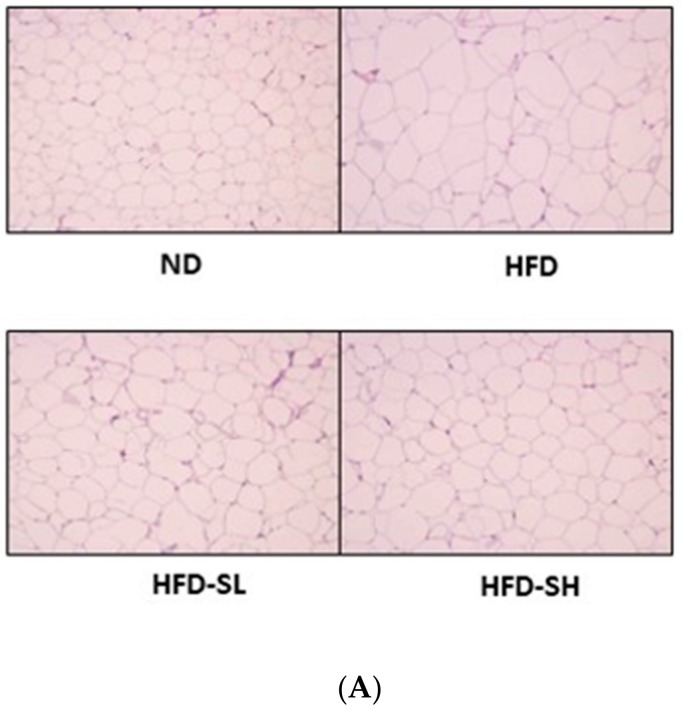

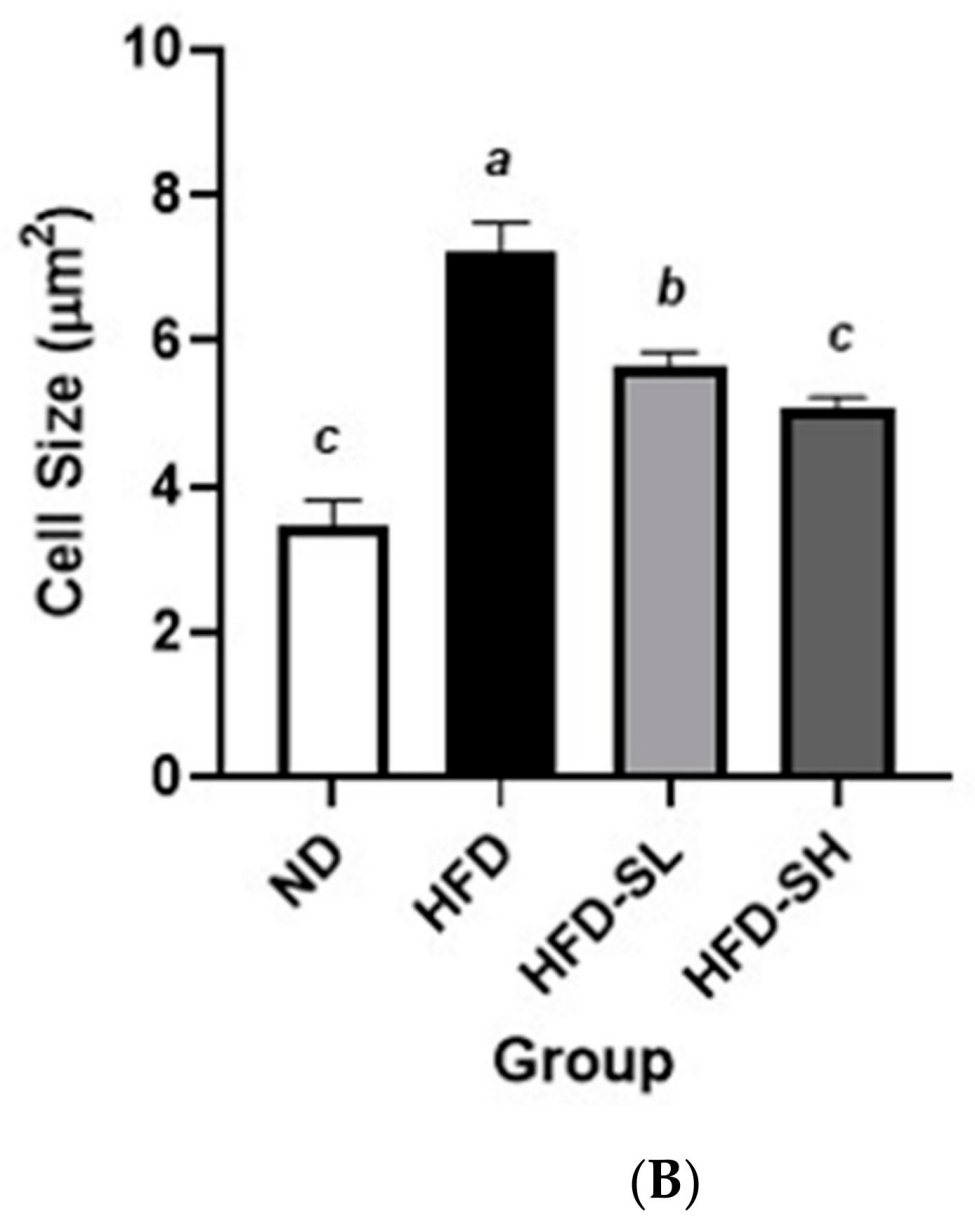
Histological analysis of epididymal adipose tissue (EAT). Rats were fed the normal diet or high-fat diet with/without *Spergularia marina* Griseb powder supplementation for 4 weeks. EAT was stained with H&E. Magnification, 100×. The surface area of EAT was measured using the Image J program. ND, Normal diet; HFD, High-fat diet; HFD-SL, High-fat diet + 3% of *Spergularia marina* Griseb; HFD-SH, High-fat diet + 5% of Spergularia marina Griseb. (**A**) Representative figures from each group. (**B**) The quantification of A. Values are means ± SDs, *n* = 8. Data were analyzed by one-way ANOVA using Kruskal–Wallis analysis followed by Tukey’s post hoc test. Means labeled without a common letter differ, *p* < 0.05.

To determine the lipid accumulation levels in the liver tissue histologically, Oil Red O staining was performed. Notably, the SMP supplemented groups (HFD-SL, HFD-SH) demonstrated inhibited lipid accumulation in the liver tissues in comparison to the HFD group (Figure 4). The EAT samples were stained with H&E staining to measure cell size; the cells were observed under the microscope and analyzed using an image analyzer (Figure 5A). Histological analysis of adipose tissues in the HFD group demonstrated a larger cell size in EATs than that of the ND group. In a dose-dependent manner, the SMP supplemented groups demonstrated a decreased adipocyte size compared to the HFD group (Figure 5B). Hence, the results suggested that reduced BW gain could be attributed to the decreased fat accumulation in the adipocytes [31]. Furthermore, the results indicated that SMP supplementation could improve the lipid profiles in the serum, liver, and adipose tissue, and could decrease lipid accumulation in the liver and adipose tissues.

## 4. Discussion

Obesity is the induced hyperplasia and/or hypertrophy of adipocytes [9]. Hyperplasia of adipocytes is regulated by the proliferation and differentiation of cells, whereas hypertrophy of adipocytes is controlled by the balance between lipogenesis and lipolysis [10]. To elucidate the underlying mechanism and the treatment of obesity, it is crucial to investigate lipid accumulation and lipolysis in cells. The 3T3-L1 cell line used in the current study is the most widely used for evaluating antiobesity activity [32]. Therefore, the study of adipogenesis in 3T3-L1 is important to understand obesity. Furthermore, the regulation of adipogenesis can be one strategy in the treatment and management of obesity.

Halophytes are salt-tolerant plants growing in saline environments and can be found on the western coast of the Korean peninsula. Reportedly, halophytes are known to have various secondary metabolism and adaptive mechanisms against severe environments, such as salt stress, compared to terrestrial plants [33]. Additionally, halophytes contain a high content of essential minerals, amino acids, phenolic acids, and flavonoids [14]. Several species of *Spergularia* have been examined and have reported beneficial effects on human health. These beneficial effects include antidiabetic [16], hypoglycemic [34], diuretic [35], and cholesterol-lowering [36] effects. For decades, *Spergularia marina* (‘Sebalnamul’ in Korean), a local food preference in South Korea, has been regarded as a nutritious source of amino acids, vitamins, and minerals; however, few studies have investigated the regulated physiological factors in vitro and in vivo [37,38,39].

The present study, for the first time, demonstrated that SME induced a significant inhibition of 3T3-L1 adipocyte proliferation, differentiation, lipid accumulation, and lipolysis (Figure 1). Furthermore, while 3T3-L1 pre-adipocytes are a widely accepted model for obesity research, their results cannot be sufficiently and directly extrapolated to human physiology, given the physiological and metabolic differences between species [18]. Porcine pre-adipocytes are a superior model for the study of adipogenesis and obesity-related diseases compared to rodent cell models owing to their higher similarity to human cells [40]. Hence, primary cultures of porcine pre-adipocytes have served as a useful tool for investigating pre-adipocyte proliferation and differentiation [29]. We performed adipocyte proliferation and differentiation and assessed TG accumulation using porcine pre-adipocytes. With regards to cell proliferation, differentiation, TG accumulation and lipolysis, SME demonstrated a similar trend to the results observed in the 3T3-L1 cells. (Figure 2). GPDH is an enzyme that converts dehydroxyacetone phosphate to glycerol-3-phosphate and is useful to investigate the differentiation of pre-adipocytes into mature adipocytes [41]. In this study, treatment with different concentrations of SMEs inhibited the GPDH acidity in 3T3-L1 cells and porcine pre-adipocytes in a dose-dependent manner (Figure 1B and Figure 2B). The decreased TG accumulation demonstrated with SME treatment in adipocytes (Figure 1C and Figure 2C) reflected the less differentiated adipocytes. LPL is an important enzyme in regulating lipid accumulation and lipid mobilization and is expressed early during the differentiation of the cell [42]. Consistently, LPL activities in adipocytes (Figure 1D and Figure 2D) were reduced with the treatment of 100 and 200 μg/mL SME. Similarly, the ethanolic extract (50%) from *Salsola komarovi* inhibited TG accumulation in a dose-dependent manner [43]. Kim et al. reported that 3T3-L1 cells treated with polymannuronate extracted from *Phaephycease* (brown algae) showed a reduction in GPDH activity and inhibited adipocyte differentiation [44]. The measurement of the TG content in cells is one method to evaluate differentiation of adipocyte [45]. Collectively, these results suggest that SME may block mammalian adipogenesis.

HFD induced lipid accumulation in the liver, resulting in liver weight gain [46], and it is well known that HFD induces an increase in WAT weight. The liver is the main organ to play an important role in lipoprotein biosynthesis and distribution [47]. In this study, HFD did not increase the weight of the liver tissue. Additionally, SMP supplementation did not significantly regulate liver tissue weight (Table 3). Hepatic TG and TC levels were significantly increased in the HFD group compared to the ND group. Hepatic TG levels were significantly reduced in the HFD-S groups compared to the HFD group (Table 6). The decreased TG contents were confirmed by the observation of smaller lipid droplets in the liver tissue following the supplementation of SMP (HFD-SL, HFD-SH) (Figure 4). Therefore, SM might play a protective role in the liver by inhibiting excessive lipid accumulation. Among adipose tissues, EAT weight was significantly reduced in the HFD-SL group in comparison to the HFD group (Table 3). Similarly, we observed that the HFD group demonstrated higher TG and TC levels in EAT and MAT compared to the ND group. TG levels in EAT showed a tendency to reduce in the HFD-SL group compared to the HFD group, with the higher SMP supplement reducing the levels of TC in EAT (Table 6). Furthermore, the higher supplement of SMP (HFD-SH) prevented the elevation of TG and TC levels in MAT induced by HFD feeding. The increased size of adipocytes in the HFD-induced obese rats indicates that the accumulation of TG in adipocytes was due to the consumption of HFD. Adipocyte size measurement is one of the methods to assess obesity [48]. In this study, adipocyte size in WAT significantly decreased with SMP supplementation under HFD feeding, implying that adipocyte hypertrophy was prevented due to the inhibition of TG accumulation (Figure 5). These results suggested that ameliorated obesity by SM could be attributed to the reduced adiposity in WAT, resulting in improved lipid accumulation. Relatively higher contents of dietary fiber in SMP (4.45%; data not shown) supplementation may attenuate systemic and peripheral fat accumulation markedly against high fat consumption in rodents. Recent reports addressed that enriched fiber consumption was also effective to decrease systemic and hepatic lipids levels significantly against dietary high fat consumption in mammals [49,50].

Serum ALT and AST are enzymatic markers of liver damage from fatty liver caused by HFD, high cholesterol diet, and alcohol, or damage of liver tissue. These pathological conditions accelerate the release of these enzymes into the blood, leading to higher activities [51]. The current results showed that the SMP supplement reduced ALT and AST activities in serum from the HFD-induced obese rats (Table 4). In addition, LDH is an enzyme involved in the oxidation–reduction reaction at the final step of anaerobic glycolysis in the cell. It has been reported that LDH activity markedly increased in acute hepatitis, early liver cancer, myocardial infarction, pernicious anemia, and leukemia [52]. In this study, in serum LDH activity elevated by HFD, a decreased trend was observed in the SMP-supplemented groups. Abdominal adiposity is strongly related to insulin resistance, impaired glucose metabolism, eventually leading to the development of type 2 diabetes mellitus (T2DM) [53]. Therefore, we measured blood glucose in this setting and observed that the blood glucose level was significantly increased following HFD feeding. Notably, the SMP supplement reduced glucose levels in a dose-dependent manner (Table 5). This indicates that HFD feeding increased the risk of metabolic syndrome and possibly diabetes. However, to ascertain results, insulin resistance should be carefully evaluated; one of the representative methodologies is the oral glucose tolerance test (OGTT) [54]. 

HDL-C and LDL-C are lipoproteins that play a role in the transfer of cholesterol. HDL-C is considered beneficial because it removes excess cholesterol from tissues and carries it to the liver for disposal. Dyslipidemia is characterized by elevated plasma cholesterol, especially increased LDL-C levels. In contrast to HDL-C, LDL-C is undesirable as it deposits the excess cholesterols in blood vessels, inducing and contributing to cardiovascular disease [55]. In addition, the accumulation of abdominal fat demonstrates higher TG levels in blood [56]. In this study, SMP supplementation indicated a tendency to increase HDL-C, compared to the HFD group, but was not statistically significant. On the other hand, LDL-C was inhibited in SMP supplemented groups in comparison to the HFD group (Table 5).

There are several reports using halophytes to treat HFD-induced obesity and metabolic diseases. *Salicornia herbacea* L. (glasswort) [57,58,59], *Salsola komarovi* [34], *Salicornia herbacea* L. [60], *Limonium tetragonum* [61], *Oenanthe javanica* [62], and *Nitraria retusa* [63] reported antiobesity effects on BW reduction and improved serum lipids profiles. In addition, *Salicornia herbacea* L. was reported that a high cholesterol diet resulted in a significant elevation of serum ALT and AST activities. Notably, the enzymatic hydrolysate of glasswort decreased ALT activity [64]. These data further illustrate the efficacy of halophytes as physiological functional food.

Previously, it has been reported that the decrease in serum lipids (TG, TC, and LDL-C) reduced the risk of CVD. It is well documented that the metabolic syndrome is associated with an increased risk of all-cause mortality and CVD [65]. In this study, we observed that SMP supplementation improved AI and CRF (Table 5), implying that the supplement could be a preventive treatment in metabolic-syndrome-related CVD. Hence, it is essential to measure cardiac function by performing echocardiography along with histological evaluations [66]. In addition, we need further studies as to whether SM treatment could attenuate the size of fully differentiated adipocyte and relevant metabolic complications to evaluate the effects of SMP in adulthood obesity since, in this study, we mainly focused the antiadipogenic effect of SM. Moreover, in our settings, we could not clearly demonstrate the potential toxicity of SM due to the absence group assignment for ND with SMP. However, we assume that SM is generally a safe material since Kim et al. reported that SM has significant, remarkable radical scavenging effects in murine adipocytes at the concentration of 100 μg/mL [37]. Additionally, Fatih Karadeniz et al. reported that SME treatment into pre-osteoblasts did not cause cytotoxicity at the concentration of 100 μg/mL [67]. Moreover, in our experimental animals, hepatic AST and ALT, which are elevated in toxic responses, were significantly suppressed in SMP supplementation against HFD-fed rats. Therefore, we logically postulate that SM, a traditionally edible food in Korea, may be considered as a relatively safe material in our experimental setting. Collectively, the current research proved the effects of SM, a kind of halophyte, as an antiobesity agent. Furthermore, SM improves the lipid profile and could alleviate the metabolic syndrome, including the risk of CVD.

## 5. Conclusions

This study investigated the antiobesity effects of SM in vitro and in vivo. SM exhibited antiobesity effects by modulating adipogenesis and lipolysis in the animal study. Our data suggested that *Spergularia marina* Griseb could have a favorable role in lipid metabolism in the serum, liver, and adipose tissue and may decrease the accumulation of TG.

## Figures and Tables

**Table 1:** Composition of experimental diet.

Diet Composition (g^1^)	ND ^2^	HFD ^3^	HFD-SL	HFD-SH
Casein	200	200	200	200
L-Cystine	3	3	3	3
Corn starch	399.986	299.986	269.986	249.986
Dextrose	100	100	100	100
Sucrose	50	50	50	50
Cellulose	50	50	50	50
Lard	100	200	200	200
Soybean oil	50	50	50	50
Mineral mix ^4^	35	35	35	35
Vitamin mix ^5^	10	10	10	10
Choline chloride	2	2	2	2
*tert*-Butylhydroquinone	0.014	0.056	0.056	0.056
*Spergularia marina* Griseb	0.0	0.0	30	50
Total (g)	1000	1000.042	1000.042	1000.042
Total energy (kcal)	4401.94	4901.944	4781.944	4701.944
Fat (kcal %)	30.67	45.90	47.05	47.85

^1^ g: gram. ^2^ ND: Normal diet. ^3^ HFD: High-fat diet. ^4,5^ AIN-93-MX mineral mixture and AIN-93-VX vitamin mixture.

**Table 2:** Effects of the *Spergularia marina* Griseb powder (SMP) on body weight gain, food intake and food efficiency ratio in HFD-induced obese rats.

Group	*n*	Body Weight Gain (g/day)	Food Intake (g/day)	FER (Food Efficiency Ratio)
ND	8	6.16 ± 0.52 *^c^*	21.80 ± 1.24 *^a^*	0.28 ± 0.01 *^b^*
HFD	8	8.22 ± 1.30 *^a^*	20.17 ± 2.29 *^a^*	0.41 ± 0.04 *^a^*
HFD-SL (3%)	8	7.48 ± 0.69 ^b^	19.03 ± 1.39 *^b^*	0.39 ± 0.03 *^a^*
HFD-SH (5%)	8	6.81 ± 1.03 *^c^*	18.55 ± 1.05 *^b^*	0.37 ± 0.04 *^a^*

Rats were fed the normal diet or high-fat diet with/without *Spergularia marina* Griseb powder supplementation 4 weeks. The body weight and food consumption were measured. ND, Normal diet; HFD, High-fat diet; HFD-SL, High-fat diet + 3% of *Spergularia marina* Griseb; HFD-SH, High-fat diet + 5% of *Spergularia marina* Griseb. Values are means ± SDs, *n* = 8. Data were analyzed by one-way ANOVA using Kruskal–Wallis analysis followed by Tukey’s post hoc test. Means labeled without a common letter differ, *p* < 0.05.

**Table 3:** Effects of the *Spergularia marina* Griseb powder (SMP) on weights of epididymal, mesenteric, retroperitoneal, perirenal, and total adipose tissues (EAT, MAT, RAT, PAT, Total AT) in HFD-induced obese rats.

Group	*n*	Liver (g)	EAT (g)	MAT (g)	RAT (g)	PAT (g)	Total AT (g)
ND	8	11.03 ± 1.04	4.50 ± 1.14 *^c^*	3.45 ± 0.96	6.24 ± 1.46	1.66 ± 0.32 *^b^*	15.84 ± 2.17 *^b^*
HFD	8	12.34 ± 2.70	8.34 ± 2.70 *^a,b^*	4.73 ± 1.25	8.59 ± 2.12	2.63 ± 1.08 *^a,b^*	24.28 ± 6.40 *^a^*
HFD-SL (3%)	8	11.46 ± 1.11	5.73 ± 1.06 *^b,c^*	4.19 ± 0.76	7.64 ± 2.35	2.13 ± 0.60 *^a,b^*	19.68±4.36 *^a,b^*
HFD-SH (5%)	8	11.59 ± 1.14	7.91 ± 2.0 *^a,b^*	4.70 ± 1.0	8.20 ± 1.45	2.56 ± 0.66 *^a^*	23.36 ± 4.09 *^a^*

Rats were fed the normal diet or high-fat diet with/without *Spergularia marina* Griseb powder supplementation for 4 weeks. Each adipose tissue was harvested and weighted after washing with PBS. ND, Normal diet; HFD, High-fat diet; HFD-SL, High-fat diet + 3% of *Spergularia marina* Griseb; HFD-SH, High-fat diet + 5% of *Spergularia marina* Griseb. Values are means ± SDs, *n* = 8. Data were analyzed by one-way ANOVA using Kruskal–Wallis analysis followed by Tukey’s post hoc test. Means labeled without a common letter differ, *p* < 0.05.

**Table 4:** Effects of the *Spergularia marina* Griseb powder (SMP) on the liver function.

Group	*n*	AST (U/L)	ALT (U/L)	ALP (U/L)	LDH (U/L)
ND	8	120.50 ± 20.45 *^b^*	30.75 ± 6.84 *^b^*	650.3 ± 62.0 *^b^*	687.1 ± 116.8 *^b^*
HFD	8	189.38 ± 44.39 *^a^*	61.75 ± 4.50 *^a^*	905.8 ± 109.5 *^a^*	888.4 ± 32.9 *^a^*
HFD-SL (3%)	8	153.38 ± 31.50 *^b^*	45.25± 6.65 *^a,b^*	874.9 ± 126.2 *^a^*	804.4 ± 156.8 *^a,b^*
HFD-SH (5%)	8	136.25 ± 34.74 *^b^*	38.75 ± 4.03 *^b^*	809.0 ± 91.0 *^b^*	736.1 ± 153.5 *^a,b^*

Rats were fed the normal diet or high-fat diet with/without *Spergularia marina* Griseb powder supplementation for 4 weeks. Aspartate aminotransferase (AST), alanine aminotransferase (ALT), alkaline phosphatase (ALP), and lactate dehydrogenase (LDH) were measured in serum from the experimental rats. ND, Normal diet; HFD, High-fat diet; HFD-SL, High-fat diet + 3% of *Spergularia marina* Griseb; HFD-SH, High-fat diet + 5% of *Spergularia marina* Griseb. Values are mean ± SD, *n* = 8. Data were analyzed by one-way ANOVA using Kruskal–Wallis analysis followed by Tukey’s post hoc test. Means labeled without a common letter differ, *p* < 0.05.

**Table 5:** Effects of the *Spergularia marina* Griseb powder (SMP) on the serum lipids profiles in HFD-induced obese rats.

Group	*n*	HDL-C (mg/dL)	TC (mg/dL)	TG (mg/dL)	Glu (mg/dL)	LDL-C (mg/dL)	AI	CRF
ND	8	65.13 ± 9.42*^a^*	75.88 ± 8.66*^b^*	64.75 ± 16.12*^b^*	131.5 ± 16.6 *^c^*	24.70 ± 7.08*^c^*	0.17 ± 0.11*^b^*	1.17 ± 0.11*^b^*
HFD	8	47.63 ± 7.05*^b^*	101.63 ± 12.92*^a^*	111.75 ± 25.08*^a^*	179.4 ± 12.5 *^a^*	76.35 ± 12.45*^a^*	1.15 ± 0.24*^a^*	2.15 ± 0.24*^a^*
HFD-SL (3%)	8	51.00 ± 5.71*^a,b^*	85.75 ± 16.24*^a,b^*	76.25 ± 8.41*^a,b^*	159.3 ± 11.5*^a,b^*	50.00 ± 20.02*^a,b^*	0.71 ± 0.43*^b^*	1.71 ± 0.43*^b^*
HFD-SH (5%)	8	54.25 ± 13.13*^a,b^*	77.88 ± 10.62*^a,b^*	65.63 ± 13.79*^b^*	148.9 ± 9.3*^b,c^*	36.75 ± 18.81*^b,c^*	0.51 ± 0.42*^b^*	1.51 ± 0.42*^b^*

Rats were fed the normal diet or high-fat diet with/without *Spergularia marina* Griseb powder supplementation for 4 weeks. Serum levels of HDL-C, High-density lipoprotein cholesterol; TC, total cholesterol; TG, triglyceride; Glu, glucose; LDL-C, low-density lipoprotein cholesterol; AI, atherogenic index; CRF and cardiac risk factor were measured after isolation of serum from rats. ND, Normal diet; HFD, High-fat diet; HFD-SL, High-fat diet + 3% of *Spergularia marina* Griseb; HFD-SH, High-fat diet + 5% of *Spergularia marina* Griseb. Values are means ± SDs, *n* = 8. Data were analyzed by one-way ANOVA using Kruskal–Wallis analysis followed by Tukey’s post hoc test. Means labeled without a common letter differ, *p* < 0.05.

**Table 6:** Effects of the *Spergularia marina* Griseb powder (SMP) on the lipids in liver and adipose tissues.

Group	*n*	Liver TG (mg/g)	Liver TC (mg/g)	EAT TG (mg/g)	EAT TC (mg/g)	MAT TG (mg/g)	MAT TC (mg/g)
ND	8	25.8 ± 3.8*^c^*	82.8 ± 10.0*^b^*	88.2 ± 8.3*^c^*	75.3 ± 12.9*^c^*	35.5 ± 2.5*^c^*	25.3 ± 3.9*^c^*
HFD	8	60.0 ± 12.7*^a^*	107.9 ± 18.4*^a^*	107.3± 12.2*^a^*	125.4 ± 15.5*^a^*	62.7 ± 2.7*^a^*	39.8 ± 4.5*^a^*
HFD-SL (3%)	8	45.0 ± 12.7*^a,b^*	98.0 ± 11.7*^b^*	92.3 ± 4.7*^b,c^*	103.7±16.2*^a,b^*	44.3 ± 3.6*^a,b^*	32.2 ± 4.5*^a,b^*
HFD-SH (5%)	8	41.0 ± 7.4*^b,c^*	97.6 ± 11.7*^b^*	97.0± 13.5*^a,b^*	93.5 ± 6.4*^b,c^*	42.4 ± 4.5*^b,c^*	29.5 ± 2.7*^b,c^*

Rats were fed the normal diet or high-fat diet with/without *Spergularia marina* Griseb powder supplementation for 4 weeks. Triglycerides (TG) and total cholesterol (TC) were analyzed from the liver, epididymal adipose tissues (EAT) and mesenteric adipose tissues (MAT). ND, Normal diet; HFD, High-fat diet; HFD-SL, High-fat diet + 3% of *Spergularia marina* Griseb; HFD-SH, High-fat diet + 5% of *Spergularia marina* Griseb. Values are means ± SDs, *n* = 8. Data were analyzed by one-way ANOVA using Kruskal–Wallis analysis followed by Tukey’s post hoc test. Means labeled without a common letter differ, *p* < 0.05.
